# Left Ventricular Diastolic Dysfunction in Type 2 Diabetes—Progress and Perspectives

**DOI:** 10.3390/diagnostics9030121

**Published:** 2019-09-17

**Authors:** Elena-Daniela Grigorescu, Cristina-Mihaela Lacatusu, Mariana Floria, Bogdan-Mircea Mihai, Ioana Cretu, Laurentiu Sorodoc

**Affiliations:** 1 Diabetes, Nutrition and Metabolic Diseases, “Grigore T. Popa” University of Medicine and Pharmacy, 700115 Iași, Romaniabogdan.mihai@umfiasi.ro (B.-M.M.); 2 “Sf. Spiridon” Emergency Hospital, 700111 Iași, Romania; floria_mariana@yahoo.com (M.F.); laurentiu.sorodoc@gmail.com (L.S.); 3 Internal Medicine, “Grigore T. Popa” University of Medicine and Pharmacy, 700115 Iași, Romania; 4 Department Preventive Medicine and Interdisciplinarity, “Grigore T. Popa” University of Medicine and Pharmacy, 700115 Iași, Romania; ioana.cretu@umfiasi.ro

**Keywords:** type 2 diabetes, echocardiography, left ventricular diastolic dysfunction, E/e’ index, diabetic cardiomyopathy

## Abstract

In-depth understanding of early cardiovascular manifestations in diabetes is high on international research and prevention agendas given that cardiovascular events are the leading cause of death for diabetic patients. Our aim was to review recent developments in the echocardiographic assessment of left ventricular diastolic dysfunction (LVDD) as a telltale pre-clinical disturbance preceding diabetic cardiomyopathy. We analyzed papers in which patients had been comprehensively assessed echocardiographically according to the latest LVDD guidelines (2016), and those affording comparisons with previous, widely used recommendations (2009). We found that the updated algorithm for LVDD is more effective in predicting adverse cardiovascular events in patients with established LVDD, and less specific in grading other patients (labelled “indeterminate”). This may prove instrumental for recruiting “indeterminate” LVDD cases among patients with type 2 diabetes mellitus (T2DM) in future screening programs. As an interesting consideration, the elevated values of the index E/e’ can point to early diastolic impairment, foretelling diabetic cardiomyopathy. Identifying subclinical signs early makes clinical sense, but the complex nature of T2DM calls for further research. Specifically, longitudinal studies on rigorously selected cohorts of diabetic patients are needed to better understand and predict the subtle, slow onset of cardiac manifestations with T2DM as a complicating backdrop.

## 1. Introduction

The undisputed bidirectional relationship between type 2 diabetes mellitus (T2DM) and cardiovascular disease (CVD) acts as a vicious circle, whereby the former heightens the risk of the latter, and the latter is an important complication, comorbidity, and mortality factor in the former [1,2]. This is all the more worrying as both are increasingly prevalent worldwide. The latest available systematic review of research from across the world (57 papers on approximately 4.5 million T2DM patients over a period of 10 years) concluded that almost a third of type 2 diabetic patients also suffer from some form of CVD. Moreover, CVD was identified as the cause of death in half of the studied patients [3].

Type 2 diabetic patients may develop underlying CVD without experiencing or recognizing the telltale signs and symptoms until too late. Subclinical manifestations are therefore difficult to study and report, which makes the real scale of the problem an important question in an otherwise well-understood associated pathology. There is, however, progress: researchers have proposed appropriate terminology as well as the need for early screening tools for confirmed diabetic subjects without cardiovascular symptoms [4,5]. For instance, the concept of “unrecognized diabetic cardiac impairment” was put forward to not only include atypical, silent manifestations visible on the rest electrocardiogram (ECG), but also left ventricular diastolic dysfunction (LVDD). The latter requires more thorough imagistic investigation and, if it not detected in time, can develop into life-threatening heart failure with preserved ejection fraction [4]. This adds interest and value to early screening measures, which could become standard practice, as recommended in recent guidelines [6,7]. However, the up-scaling of adequate screening programs to include the entire population of diabetic patients at risk of CVD are yet to be made more accessible and cost efficient [8]. Despite such issues, LVDD is the easier impairment to diagnose from the wider range of early subclinical cardiac complications in type 2 diabetes. The early echocardiographic diagnosis of LVDD is of substantial practical significance in these patients, and a review of the new relevant echocardiographic criteria and other updates is thus justified.

## 2. Materials and Methods

**Figure 1:**
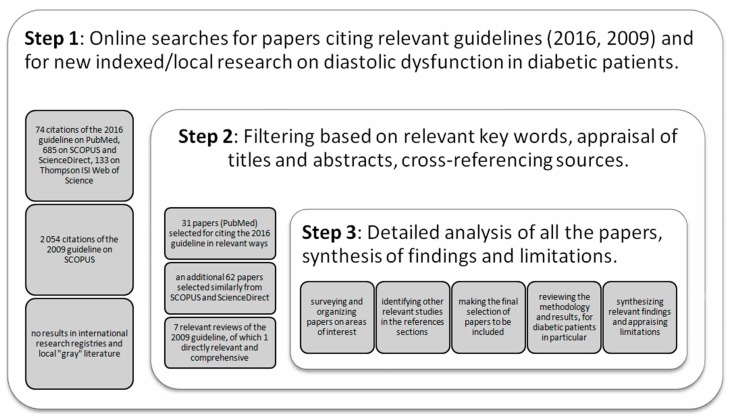
Flow chart for the study.

The most widely known, applied, and reviewed set of recommendations for assessing diastolic function is the guideline for the evaluation of left ventricular diastolic function by means of echocardiography, available since 2009 [9]. A revised version has since been issued in 2016, aiming to improve effectiveness in clinical practice [10]. In our literature search, we focused mainly on identifying published studies in which the patient data had been analyzed in accordance to these latest recommendations about LVDD from 2016. We used a protocol recommended for medical reviews and surveyed four well-established international literature databases: PubMed, Scopus, Science Direct, and Thompson ISI Web; and the flow chart for selected papers is presented in Figure 1 [11].

We started from the online text of the 2016 recommendations and investigated all its PubMed citations by mid-March 2019. There were seventy-four papers in English citing the new guidelines from a range of medical perspectives. The titles and abstracts were screened to select those papers relevant for the diagnosis, classification, and treatment of LVDD. Thirty-one papers were selected based on their use and/or appraisal of the new algorithm, the inclusion of diabetic patients in the studied cohorts, and the implications for clinical practice, etc.

Notably, only one study was found in this manner to specifically address LVDD in T2DM subjects [12]. However, upon analyzing the other selected papers, we found more research that had included data from diabetic patients [13]. Furthermore, whenever we found a study to be of partial interest, we surveyed the full text for mentions of LVDD in diabetes mellitus and pursued the subsequent bibliographic references, thus identifying 12 additional papers.

The same approach was then repeated for SCOPUS and Science Direct, resulting in 685 citations. These were filtered down to 187 based on the keywords “diastolic dysfunction” and “diabetes mellitus”. The screening of their titles and abstracts led to a further reduction to 62 papers other than those previously indexed on PubMed. Further, the 133 citations available through the Thompson ISI Web of Science search engine were analyzed, with no additional results. Finally, two international research registries did not enlist any relevant studies [14,15].

Apart from the published studies applying the 2016 algorithm on diabetic patients, we also sought to identify the most comprehensive reviews of prior research using the 2009 guidelines. On SCOPUS, the 2009 recommendations were cited 2054 times by mid-March 2019, of which seven took into account LVDD in diabetes mellitus.

## 3. Left Ventricular Diastolic Dysfunction

### 3.1. Metabolic and Structural Changes

Systolic dysfunction associated with left ventricular ejection fraction reduction was proven to be insufficiently indicative of heart failure diagnoses, as symptoms may also occur with mid-range as well as preserved ejection left ventricular fractions (recently redefined as ≥ 50%) [16]. In this context, diastolic dysfunction is a more useful indicator of early heart failure if the patient is not experiencing any symptoms, and standard ejection fraction assessment does not raise any red flags [16].

In addition, taking diastolic dysfunction into account allows for a more accurate prognosis of heart diseases other than endo- or pericardial (where different mechanisms are involved). In a nutshell, this is because diastolic dysfunction betrays the structural deterioration taking place at the level of myocardial cells and matrix. Basically, the myocardial stiffness resulting from collagen damage, interstitial fibrosis, and inflammation delays the relaxation period with negative consequences further down the chain of the diastolic and filling pressure [17,18,19,20].

**Figure 2:**
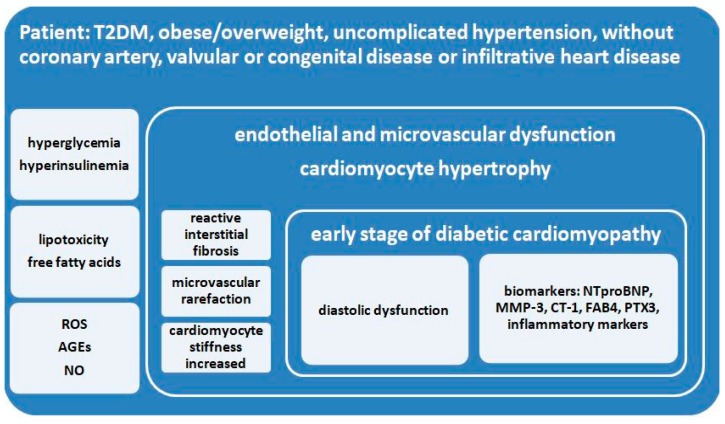
The pathways of left ventricular diastolic dysfunction in diabetic patients. AGEs: advanced glycation end-products; CT-1: cardiotrophin-1; FAB4: fatty acid-binding protein 4; MMP-3: matrix metalloproteinase-3; NO: nitric oxide; NTproBNP: N-terminal pro brain natriuretic peptide; PTX3: pentraxin-3; ROS: reactive species of oxygen.

It is important to identify metabolic and structural changes in the heart as early as possible because, in time, they bring about the progressive impairment of cardiac structure and function leading up to irreversible heart failure (HF; Figure 2). The known mechanisms and pathways involved in early cardiomyocytic damage and subsequent aggravation are metabolic disturbances (decreased glucose oxidation, increased free fatty acids), impaired cellular function (inadequate calcium handling, augmented oxidative stress, mitochondrial dysfunction), structural alterations (advanced glycation end-product, cardiomyocyte hypertrophy), in addition to the activation of the renin-angiotensin-aldosterone system (fibrosis and cardiomyocyte stiffness) and cardiac autonomic neuropathy. These are triggered by hyperglycemic and lipotoxic anomalies related to insulin resistance, the prominent feature in type 2 diabetes [21,22,23,24,25,26,27,28,29,30]. Figure 2 summarizes the cellular, structural, and functional anomalies, the main imaging features, and the serological biomarkers involved in the earlier stages of diabetic cardiomyopathy [23,25,31,32,33,34,35,36,37].

Research has been fruitful in unraveling the more subtle metabolic and structural mechanisms involved in diabetic cardiomyopathy, but important challenges still remain. For one, since diabetes-related cardiomyopathy was studied mostly in patients with a long history of diabetes, the current definition of cardiomyopathy does not address early, insidious, subclinical pathophysiological alterations. These are now generating substantial scientific interest, but existing datasets do not hold all the necessary information. More prospective studies are needed to take a new understanding of relevant molecular processes and signaling pathways from research on mice to human cohorts. Moreover, it is difficult to single out the individual contributions of hyperglycemia or of hyperinsulinemia (secondary to insulin resistance and specific to type 2 diabetes) from other risk factors such as hypertension, obesity, or coronary heart disease, because most diabetic patients enrolled in clinical trials feature a combination of these comorbidities [32,35,38,39,40]. Low levels of anti-inflammatory molecules from the sirtuin family (SIRT1, SIRT6) have recently been measured in the abdominal adipose tissue of obese, CVD-free, pre-diabetic patients, concurrent with high levels of inflammatory markers. However, according to our searches, data supporting a relationship between low levels of sirtuins and diastolic dysfunction in type 2 diabetes patients are yet to be reported in the literature, as it has already been done with regard to low SIRT1 and altered values of the myocardial performance index [41,42].

When such limitations are resolved, the scientific and clinical community will be in a better position to expand and optimize early diagnostic and therapeutic approaches. At present, various techniques using biomarkers and imagistic assessment methods can detect subclinical manifestations involved in cardiac dysfunction before it reaches more advanced, overt stages. A practical guide comprehensively pooling high-sensitivity, high-specificity approaches together would maximize these opportunities for early subclinical sign detection, prevention, and possibly even reversal in the target population [23,32,35,37].

**Figure 3:**
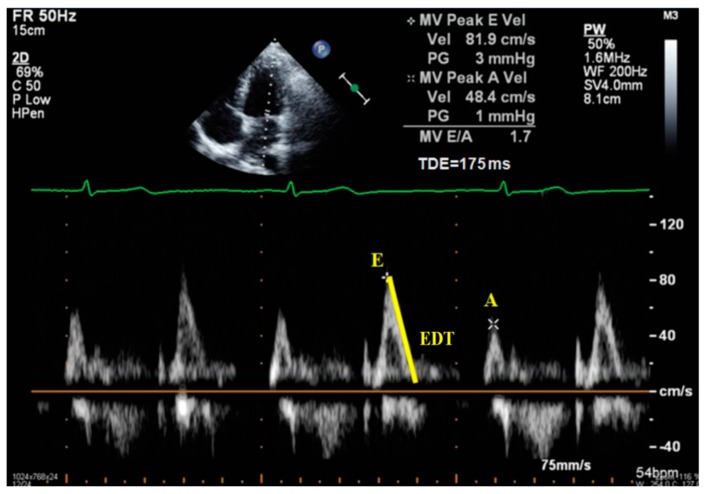
Echocardiographic assessment of mitral flow with pulsed Doppler in a patient in sinus rhythm. A: A wave velocity (cm/s); E: E wave velocity; EDT = E wave deceleration time.

The mechanisms outlined above help define a restrictive phenotype of cardiomyopathy in diabetes and are also consistent with the imagistic pattern of heart failure with preserved ejection fraction (HFpEF). However, the ejection fraction commonly used to diagnose heart failure is not a reliable indicator of cardiomyopathy in type 2 diabetes because it may still be preserved even if the LVDD has already set in (and is often present in T2DM). More elaborate imagistic measurements are needed in addition to the usual ratio between E and A waves (E/A) (Figure 3) in order to adequately assess diastolic function and detect potential heart impairment in these patients. These measurements are easy to do and should be commonplace if the diabetic patient is in sinus rhythm [43,44].

### 3.2. Echocardiographic Assessment Approaches

**Figure 4:**
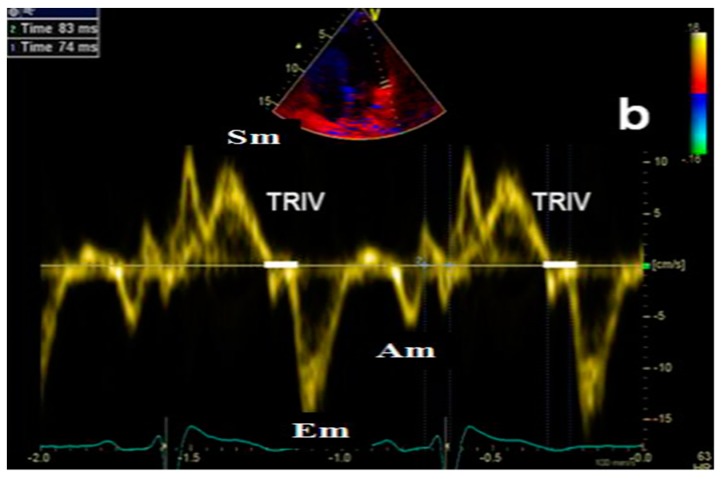
Echocardiographic assessment of isovolumetric relaxation time (IVRT) by Tissue Doppler Imaging (TDI). Am (or A’): late diastolic annular velocity (septal, lateral, and average) via tissue Doppler imaging; Em (or E’): early diastolic annular velocity (septal, lateral, and average) via tissue Doppler imaging; Sm: systolic annular velocity; TRIV: isovolumetric relaxation time.

**Figure 5:**
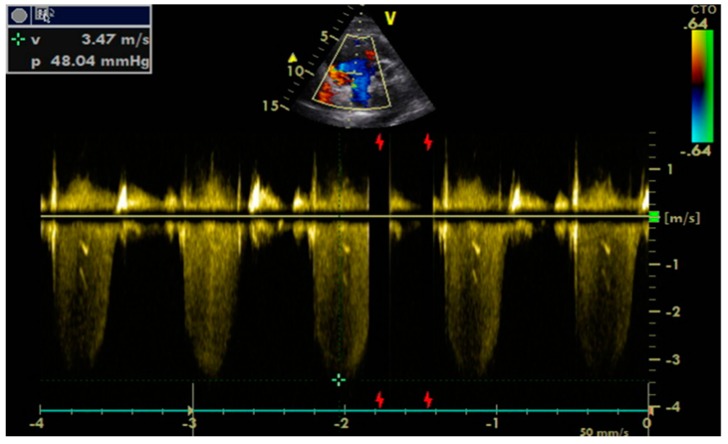
Echocardiographic assessment of tricuspid regurgitation peak velocity (TRpV) by continuous Doppler (CW) on tricuspid regurgitation jet. V: velocity; P: gradient (is 4V^2^). V: specific echocardiographic parameter (it does not mean a measurement data).

**Figure 6:**
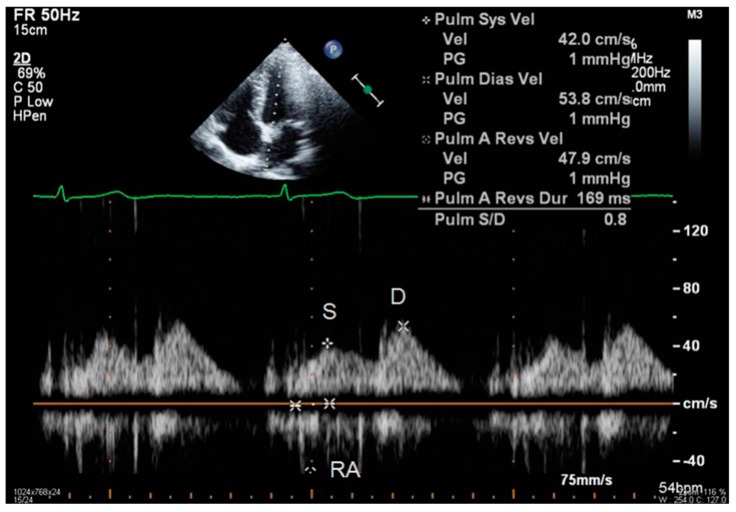
Echocardiographic measurement of S and D wave peak velocity by pulsed Doppler on right superior pulmonary vein flow. D: peak velocity of diastolic wave: RA: peak velocity of reverse atrial wave; peak S: peak velocity of systolic wave.

**Figure 7:**
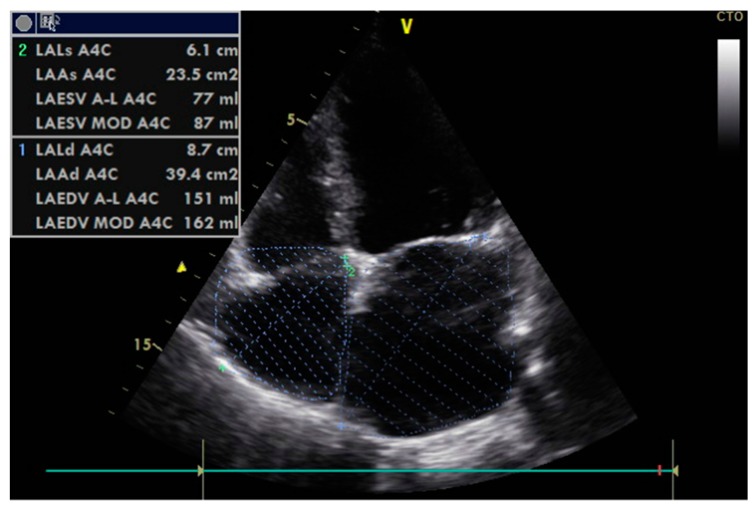
Echocardiographic measurement of left (and right) atrial volume by disks method. V: specific echocardiographic parameter (it does not mean a measurement data).

Transthoracic echocardiography is the preferred approach in assessing heart functions due to its non-invasiveness. According to the 2009 and 2016 guidelines [9,10], multiple parameters need to be measured in order to estimate left ventricular relaxation and subsequent filling pressures:

- Peak of passive filling (E wave), peak of active filling (A wave), E/A ratio, deceleration time of E wave (EDT) with pulsed Doppler (Figure 3);
- Isovolumetric relaxation time (IVRT) (Figure 4), early diastolic annular velocity E’ (septal, lateral, and average), late diastolic annular velocity A’ (septal, lateral, and average) via tissue Doppler imaging (TDI) (which measures myocardial tissue velocities during the cardiac cycle);
- Tricuspid regurgitation peak velocity (TRpV; Figure 5) with CW (continuous Doppler) on tricuspid regurgitation jet;
- S and D wave peak velocity on right superior pulmonary vein flow in pulsed Doppler (Figure 6);
- Left atrial size assessed by area or better by left atrium volume (indexed) (LAVi; Figure 7).

**Figure 8:**
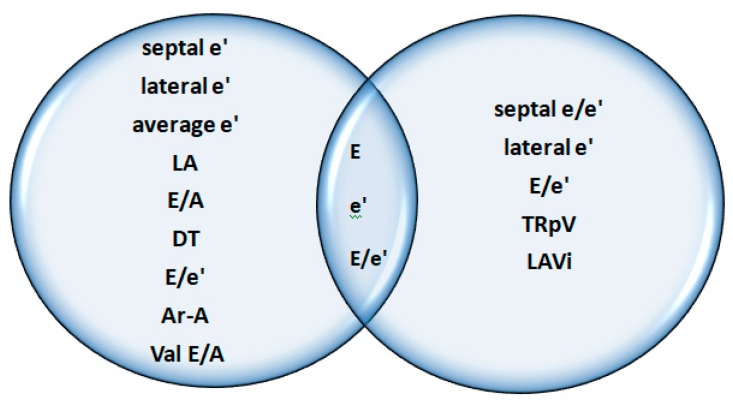
The main parameters included in 2009 and 2016 guidelines regarding LVDD. A: peak A wave velocity; Ar-A: difference between duration of A wave flow reversal and duration of the mitral A wave; E: peak E wave velocity; e’: early diastolic annular velocity e’ (septal, lateral, and average); E/A: E/A ratio; E/e’: ration between E velocity and mitral annular e’ velocity; LA left atrium; LAVi: left atrial volume indexed; DT: deceleration time of E wave; Val E/A: change in E/A with Valsalva maneuver; TRpV: peak velocity of tricuspidian regurgitation.

The 2016 guideline revised and expanded the one from 2009 with regard to how such parameters may be used to distinguish between different patterns of diastolic function when the left ventricular ejection fraction is normal (Figure 7). In 2019, the guide was further expanded in order to facilitate wider scale understanding and application in routine clinical thinking and decision-making (Figure 8) [10,45,46,47].

However, these measurements must be correctly assessed, according to the current recommendations, by experienced operators ideally accredited by national societies or the European Society of Cardiology [48].

### 3.3. Diastolic Dysfunction—Where Are We Now?

Since 2016, several researchers reviewed the differences between the two algorithms [49] and conducted comparative analyses of existing data sets containing the necessary measurements to allow processing based on the updated version (e.g., the addition of peak tricuspid regurgitation velocity for establishing the severity of diastolic dysfunction) [13,50,51]. Additional insights from studies enlisting various proportions of diabetics in their cohorts (ranging from 6% to 30%) are discussed below.

For instance, Almeida et al. looked at their 1000 subject dataset retrospectively, in which 114 were confirmed with type 2 diabetes and more than half of were at risk of diabetes. What emerged from the comparison was that the prevalence of diastolic dysfunction was considerably higher using the 2009 algorithm (38.1%), whereas it was almost negligible when assessed with the updated version (1.4%) [13]. In another retrospective analysis, a concordance of only 43% between the classifications using the two algorithms was reached, mostly with regard to normal function and mild dysfunction [51]. These findings point to the newer algorithm as potentially better suited for the assessment of moderate to severe cases, for which therapeutic management could thus be intensified. For the low grade diastolic dysfunction, the application of the algorithm may rather help with recruiting patients in screening programs.

Moreover, after monitoring 157 patients, of which 45 were diagnosed with T2DM, over the course of five years, Sanchis et al. found that the 2009 guidelines might overestimate the prevalence of first degree diastolic dysfunction, while the 2016 recommendations facilitated better prediction of cardiovascular events (similar conclusions were recently reported by Fabiani et al. in 2019) [50,52]. The latter algorithm, on the other hand, also resulted in labeling 36 patients as “indeterminate”, compared to no such cases when the former was applied [50]. Another application of the same guideline on a cohort of 235 Spanish patients with metabolic syndrome, of which 52 confirmed diabetics, resulted in a prevalence of diastolic dysfunction as low as 3%, while 58% were rated as normal and 39% were “indeterminate” before conducting further cardiopulmonary exercise tests [53].

With specific regard to patients graded as “indeterminate”, their clinical and echocardiographic characteristics seem to more closely resemble diastolic dysfunction rather than normal diastolic function. The similarities were found by Shimron et al. in a retrospective analysis of 1674 cases (20% diabetics), and they encompassed an elevated risk ratio for heart failure symptoms in both the “indeterminate” and the diastolic dysfunction groups compared to normal [54]. In our opinion, such results can inform the inclusion of “indeterminate” patients in screening programs for heart failure and justify the implementation of early therapeutic measures.

In his 2017 review, Fraser pointed out that seemingly objective and straightforward algorithms were being applied with a degree of variability depending on clinicians’ understanding and experience, creating the risk of over-, under-, and misdiagnosis. With regard to diastolic function, he noticed that the variables included in the latest algorithm accounted for a large number of combinations, leading to the impractical classification of some patients as “indeterminate”. Moreover, while imagistic technology had been perfected to provide high levels of specificity and sensitivity, there was also cognitive overload and bias undermining the effective and consistent interpretation. Consequently, Fraser urged for transdisciplinary collaboration towards automated systematization by pooling databases together and using machine learning to optimize both processes and results [55].

One interesting example of such an undertaking is by Tamas and Nylander who, in 2016, created an automated assessment process and compared its performance to two rounds of manual interpretation of the same data at different times. The inter-rater agreement between manual assessments was less than moderate, and it was poor between automated and manual, which demonstrates substantial variability in how algorithms are applied by clinicians [56]. Moreover, the discussion of methodology in Bamaiyi et al. illustrates how difficult it may be to observe guidelines fully, consistently, and accurately (e.g., keeping to the recommended number of diagnostic criteria throughout) [57].

Such contributions, as well as the challenges of interpretation in clinical practice, have been acknowledged by Nagueh, the lead author of both guidelines. His advice to practitioners and researchers is to include clinical findings, conduct complete measurements, and consider specific pathological conditions when applying the algorithm in order to minimize “indeterminate” classifications [58]. Indeed, in a recent coordinated effort employing both echocardiographic and invasive means, the importance of specialized training and experience was assessed with regard to how result interpretation may vary among clinicians. The inter-observer agreement was found to be good especially when the procedure is conducted in accordance with the 2016 guidelines and the data are carefully measured [59]. In our view, such validation by invasive methods and reliability in practice makes for a compelling argument in favor of mainstreaming this approach.

With regard to the need and opportunity for preventive screening, until recently, patients with diabetes were not among those for whom such programs were recommended [60]. However, a recent prospective study used the 2016 guidelines to identify diastolic dysfunction in 47% of the 219 diabetic patients without cardiovascular complications, who had been monitored over a period of five years. A predictive model was proposed based on clinical, ECG, and echocardiographic data. When diastolic dysfunction was assessed and computed into the model, the power to predict the risk of adverse events improved [61]. Thus, it may be worth devising screenings of diabetic subjects who meet certain criteria but do not yet experience the clinical symptoms of heart failure, in order to prevent related incidents. Some hurdles yet to overcome are, first, to assess the cost-effectiveness of such programs, and, second, to allocate the necessary resources.

Another perspective that has been considered is the extent to which diabetes per se, versus in association with other factors, can exert an aggravating influence on the diastolic function of the patient. For instance, while an increased body mass index (BMI) (overweight and obesity) was found capable of worsening systolic and diastolic function independently [62], it seems that diabetes can act in a similar fashion; and, importantly, in the presence of both, negative effects accumulate [63]. Likewise, diabetes was proven to distort diastolic function both together with and independently from associated hypertension, which is already known to alter the left ventricle [64].

Moreover, the mean E/e’ ratio was found in several studies to be higher in diabetic patients, including those who do not exhibit overt cardiovascular manifestations (see Zopinni et al.’s meta-analysis of 15 cross-sectional studies, published in 2018). The question is whether or not an elevated E/e’ index is an indication, or even a “hallmark”, of early diastolic alteration in diabetic cardiomyopathy. However, not everyone is convinced of this assertion based on research pointing to modifications occurring possibly even before the onset of diastolic impairment [65]. In our experience, a more comprehensive picture of diabetes-induced cardiac modifications, which are slow to unfold, may be better achieved by assessing the E/e’ ratio not alone but in combination with LAVi. As a volumetric parameter, LAVi is less susceptible to the patient’s heart physiology at the time of measurement because it is a consequence of chronic diastolic dysfunction [66].

### 3.4. Additional Remarks

#### 3.4.1. General Patient Information

When interpreting echocardiographic data using algorithms for classification of diastolic dysfunction, the demographic profile of patient cohorts may bear relevance. For instance, D’Andrea et al. applied the 2009 and 2016 algorithms to a healthy population of 1168 Caucasians and found statistically significant E/e’ differences in men versus women. In addition, this index appeared to also naturally increase with age [67] without it being a prognostic indicator of cardiovascular outcomes, since the study was conducted on healthy individuals [68]. Tamas and Nylander took this a step further by proposing actual correction values based on age when using the 2016 algorithm to interpret a patient’s echocardiographic results [56].

A similar argument can be made about anthropological characteristics such as height and weight. These are computed into the Body Surface Area (BSA), which is then used to determine the Left Atrial Volume index (LAVi), one parameter included in the assessment of diastolic function. As an example, healthy native Dutch people are generally taller and, consequently, they have higher BSAs. When assessing left atrial parameters and indices in Dutch patients, van Grootel et al. suggest that normative values be adjusted accordingly [69].

#### 3.4.2. Alternative Diagnostic Methods

Myocardial properties susceptible to diastolic dysfunction can nowadays be investigated non-invasively in a variety of ways. They include mechanical deformation (by speckle tracking), fibrosis and ischemia (by magnetic resonance imaging), autonomic dysfunction (by nuclear imaging), and filling pressure during physical exertion (by exercise stress testing) [35,66,70,71,72,73]. However, the potential for these innovative methods to reach far and wide and facilitate early detection is inhibited by their substantial costs and complexity—a further need for standardization—and, in some cases, even health hazards (e.g., radiation).

#### 3.4.3. Treatment Spin-Offs

There is consensus on both sides of the Atlantic regarding the management of hyperglycemia required in case of already established atherosclerotic cardiovascular disease [74]. However, for patients with diabetes more broadly at risk of developing cardiac pathology, the respective guidelines are less prescriptive. Certain medication aimed at lowering blood glucose levels in diabetic patients has also been found to provide cardiovascular benefits. Expert reviewers have noted that diabetes is capable of exerting a degree of influence on the pathological mechanisms leading to heart failure which, in its early stage, entails diastolic dysfunction. This relationship may explain why cardiac benefits were acknowledged in diabetic patients treated with novel antihyperglycemic medication such as sodium-glucose cotransporter-2 inhibitors and Glucagon-like peptide-1 receptor agonists [1,24,75,76,77,78,79]. Further studies are being undertaken with regard to maximizing therapeutic outcomes [80].

## 4. Limitations of The Study

Note should be made that this is a narrative review and that we considered the possibility of including more studies from before 2016, ultimately deciding against it. These would have introduced too much heterogeneity in the underlying conceptual framework of definitions and criteria, as Selmeryd et al. and Bouthoorn et al. had already encountered in their reviews of studies from before the publication of the 2016 LVDD algorithm [81,82]. Similarly, in a recent meta-analysis of 15 cross-sectional studies from 2005 to 2017 regarding the E/e’ index as an updated criterion in the 2016 algorithm, the equipment used and other factors not part of the algorithm were found as sources of substantial heterogeneity [12].

Among the selected papers, some used prediction models based on echocardiographic parameters in combination with biomarkers (e.g., N-terminal pro brain natriuretic peptide (NTproBNP), which cardiomyocytes produce when subjected to mechanical stress) [83]. While we appreciate that such approaches afford a more accurate process of diagnosing systolic and diastolic heart failure, we chose not to pursue this lead. This is mainly because, in low resource countries, the costs of such tests are significant and would make wide scale screening programs prohibitively expensive. Cost-efficiency issues aside, prospective heart failure studies enrolling asymptomatic T2DM patients may help establish if myocyte stress biomarkers (BNP, ST2) can predict HFpEF-related events. High levels of these markers have already been associated with a worsening prognosis in HFrEF patients with associated metabolic syndrome and who were treated with implantable cardioverter defibrillators [84].

We are aware of the current medical interest in the understanding of molecular mechanisms that could lead to new discoveries and theories explaining the onset and development of heart failure with preserved ejection fraction in patients with type 2 diabetes. However, we took note of methodological limitations in this area (e.g., inappropriate experimental designs and insufficient supply of tissue samples from human subjects) and hence our choice was to err on the side of caution [85].

## 5. Conclusions

What we have learned with regard to the diagnosis and grading of diastolic function is that the latest, 2016 guideline appears to serve best in identifying the more severe cases. Moreover, the echocardiographic parameters, such as the diastolic index E/e’, were found to be predictors of adverse cardiovascular events. At the same time, while it does not fully cater for the subclinical domain, it does create an opportunity to select the cases classified as “indeterminate” for the purpose of screening for silent CVD and subsequent monitoring.

Concurrently, upon reviewing available studies and in relation to other recent reviews, editorials, and commentaries, we have become aware of the challenges of full investigations and accurate interpretations: equipment model and availability, methodological heterogeneity, patient profiling and matching, inter-observer variability, and clinician experience and expertise. Moreover, the underlying demographic and anthropological profile of the population to which the patient belongs may further interfere with interpretation and results, in conjunction with known associated comorbidities. Such difficulties have imprinted a significant degree of heterogeneity onto existing scientific endeavors and outcomes. As guideline improvements are constantly sought, retrospective and comparative studies are useful but limited to those databases affording analyses with updated parameters and formulae.

With or without the ‘usual suspects’ in the picture (aging, obesity, metabolic syndrome, hypertension), diabetes can distort diastolic function by itself and contribute to the snowball effect of serious cardiac complications such as heart failure. In the daily clinical practice of managing the wide spectrum of complications related to diabetes, not all clinicians may fully appreciate the significance of detecting diastolic dysfunction early, or lack methodological capacity to do it. Therefore, the widespread screening of diabetic patients, aiming to detect subclinical manifestations as early as possible, makes clinical sense. Current concerns regarding the poor cost efficiency of such programs could be outweighed by more scientific evidence into the long term benefits of early detection and prevention.

While researchers have been including patients with T2DM in their cohorts, practitioners could learn more from further studies focusing exclusively on the aforementioned diabetic profile (Figure 2) via robust methodologies of matching subjects with control groups. Moreover, more longitudinal research is needed to trace the slow progression, regression, or stagnation of subclinical cardiac manifestations over longer periods of time. Such studies would require rigorous monitoring and planning in order to account for inherently confounding factors (personal, clinical, and institutional). Given the scale and complexity of the issues, technological advances in the field of big data, artificial intelligence, and online collaboration should be taken advantage of. A multi-centric, inter-disciplinary, semi-automated approach to collecting, processing, interpreting, and studying such information would, on one hand, help reduce variability and heterogeneity while, at the same time, advancing our understanding of asymptomatic CVD in type 2 diabetes to enable preventative screening.

## References

[1] Kenny H., Abel E. (2019). Heart Failure in Type 2 Diabetes Mellitus. Circ. Res..

[2] Newman J., Schwartzbard A., Weintraub H., Goldberg I., Berger J. (2017). Primary Prevention of Cardiovascular Disease in Diabetes Mellitus. J. Am. Coll. Cardiol..

[3] Einarson T., Acs A., Ludwig C., Panton U. (2018). Prevalence of cardiovascular disease in type 2 diabetes: A systematic literature review of scientific evidence from across the world in 2007–2017. Cardiovasc. Diabetol..

[4] Schernthaner G., Lotan C., Baltadzhieva-Trendafilova E., Ceponis J., Clodi M., Ducena K., Goncalvesova E., Guja C., Honka M., Janež A. (2018). Unrecognised cardiovascular disease in type 2 diabetes: Is it time to act earlier?. Cardiovasc. Diabetol..

[5] Fox C., Golden S., Anderson C., Bray G., Burke L., de Boer I., Deedwania P., Eckel R., Ershow A., Fradkin J. (2015). Update on Prevention of Cardiovascular Disease in Adults with Type 2 Diabetes Mellitus in Light of Recent Evidence: A Scientific Statement From the American Heart Association and the American Diabetes Association. Diabetes Care.

[6] American Diabetes Association (2019). 10. Cardiovascular disease and risk management: Standards of Medical Care in Diabetes—2019. Diabetes Care.

[7] Atherton J., Sindone A., De Pasquale C., Driscoll A., MacDonald P., Hopper I., Kistler P., Briffa T., Wong J., Abhayaratna W. (2018). National Heart Foundation of Australia and Cardiac Society of Australia and New Zealand: Guidelines for the Prevention, Detection, and Management of Heart Failure in Australia 2018. Heart Lung Circ..

[8] Makrilakis K., Liatis S. (2017). Cardiovascular Screening for the Asymptomatic Patient with Diabetes: More Cons Than Pros. J. Diabetes Res..

[9] Nagueh S., Appleton C., Gillebert T., Marino P., Oh J., Smiseth O., Waggoner A., Flachskampf F., Pellikka P., Evangelista A. (2009). Recommendations for the Evaluation of Left Ventricular Diastolic Function by Echocardiography. J. Am. Soc. Echocardiogr..

[10] Nagueh S.F., Smiseth O.A., Appleton C.P., Byrd B.F., Dokainish H., Edvardsen T., Flachskampf F.A., Gillebert T.C., Klein A.L., Lancellotti P. (2016). Recommendations for the Evaluation of Left Ventricular Diastolic Function by Echocardiography: An Update from the American Society of Echocardiography and the European Association of Cardiovascular Imaging. J. Am. Soc. Echocardiogr..

[11] Lang T.A., Secic M., Lang T.A., Secic M. (2006). Synthesizing the Results of Related Studies. How to Report Statistics in Medicine.

[12] Zoppini G., Bergamini C., Mantovani A., Dauriz M., Targher G., Rossi A., Bonora E. (2018). The E/e’ ratio difference between subjects with type 2 diabetes and controls. A meta-analysis of clinical studies. PLoS ONE.

[13] Almeida J., Fontes-Carvalho R., Sampaio F., Ribeiro J., Bettencourt P., Flachskampf F., Leite-Moreira A., Azevedo A. (2017). Impact of the 2016 ASE/EACVI recommendations on the prevalence of diastolic dysfunction in the general population. Eur. Heart J. Cardiovasc. Imaging.

[14] ISRCTN Registry. http://www.isrctn.com/.

[15] Research Registry. https://www.researchregistry.com/.

[16] Ponikowski P., Voors A., Anker S., Bueno H., Cleland J., Coats A., Falk V., González-Juanatey J., Harjola V., Jankowska E. (2016). 2016 ESC Guidelines for the diagnosis and treatment of acute and chronic heart failure. Eur. J. Heart Fail..

[17] Borlaug B.A., Paulus W.J. (2011). Heart Failure with Presearved Ejection Fraction: Pathophysiology, Diagnostic, and Treatment. Eur. Heart J..

[18] Frati G., Schirone L., Chimenti I., Yee D., Biondi-Zoccai G., Volpe M., Sciarretta S. (2017). An overview of the inflammatory signalling mechanisms in the myocardium underlying the development of diabetic cardiomyopathy. Cardiovasc. Res..

[19] Jia G., DeMarco V., Sowers J. (2016). Insulin resistance and hyperinsulinaemia in diabetic cardiomyopathy. Nat. Rev. Endocrinol..

[20] Mandavia C., Aroor A., DeMarco V., Sowers J. (2013). Molecular and metabolic mechanisms of cardiac dysfunction in diabetes. Life Sci..

[21] Seferović P., Petrie M., Filippatos G., Anker S., Rosano G., Bauersachs J., Paulus W., Komajda M., Cosentino F., de Boer R. (2018). Type 2 diabetes mellitus and heart failure: A position statement from the Heart Failure Association of the European Society of Cardiology. Eur. J. Heart Fail..

[22] Guja C., Dănciulescu Miulescu S. (2018). Heart failure in type 2 diabetes – the forgotten complication. Rom. J. Diabetes Nutr. Metab. Dis..

[23] Chavali V., Tyagi S.C., Mishra P. (2013). Predictors and prevention of diabetic cardiomyopathy. Diabetes Metab Syndr. Obes. Targets Ther..

[24] Maack C., Lehrke M., Backs J., Heinzel F., Hulot J., Marx N., Paulus W., Rossignol P., Taegtmeyer H., Bauersachs J. (2018). Heart failure and diabetes: Metabolic alterations and therapeutic interventions: A state-of-the-art review from the Translational Research Committee of the Heart Failure Association–European Society of Cardiology. Eur. Heart J..

[25] Pappachan J., Varughese G., Sriraman R., Arunagirinathan G. (2013). Diabetic cardiomyopathy: Pathophysiology, diagnostic evaluation and management. World J. Diabetes.

[26] Huang D., Refaat M., Mohammedi K., Jayyousi A., Al Suwaidi J., Abi Khalil C. (2017). Macrovascular Complications in Patients with Diabetes and Prediabetes. Biomed. Res. Int..

[27] Sardu C., De Lucia C., Wallner M., Santulli G. (2019). Diabetes Mellitus and Its Cardiovascular Complications: New Insights into an Old Disease. J. Diabetes Res..

[28] de Mattos Matheus A.S., Tannus L.R., Cobas R.A., Palma C.C., Negrato C.A., de Brito Gomes M. (2013). Impact of Diabetes on Cardiovascular Disease: An Update. Int J. Hypertens.

[29] Riehle C., Bauersachs J. (2019). Of mice and men: Models and mechanisms of diabetic cardiomyopathy. Basic Res. Cardiol..

[30] Wan S., Vogel M., Chen H. (2014). Pre-Clinical Diastolic Dysfunction. JACC.

[31] Low Wang C., Hess C., Hiatt W., Goldfine A. (2016). Clinical Update: Cardiovascular Disease in Diabetes Mellitus. Circulation.

[32] Gilca G., Stefanescu G., Badulescu O., Tanase D., Bararu I., Ciocoiu M. (2017). Diabetic Cardiomyopathy: Current Approach and Potential Diagnostic and Therapeutic Targets. J. Diabetes Res..

[33] Soares Felício J., Cavalcante Koury C., Tavares Carvalho C., Felício Abrahão Neto J., Barbosa Miléo K., Pontes Arbage T., Dias Silva D., Ferreira de Oliveira A., Soares Peixoto A., Bentes Figueiredo A. (2016). Present Insights on Cardiomyopathy in Diabetic Patients. Curr. Diabetes Rev..

[34] Mizamtsidi M., Paschou S., Grapsa J., Vryonidou A. (2016). Diabetic cardiomyopathy: A clinical entity or a cluster of molecular heart changes?. Eur. J. Clin. Investig..

[35] Lorenzo-Almorós A., Tuñón J., Orejas M., Cortés M., Egido J., Lorenzo Ó. (2017). Diagnostic approaches for diabetic cardiomyopathy. Cardiovasc. Diabetol..

[36] Maisch B., Alter P., Pankuweit S. (2011). Diabetic cardiomyopathy—fact or fiction?. Herz.

[37] Gajardo A., Llancaqueo M. (2018). Circulating biomarkers of left ventricular diastolic function and dysfunction: Filling the research gap under high pressure. Eur. J. Prev. Cardiol..

[38] Jia G., Whaley-Connell A., Sowers J. (2018). Diabetic cardiomyopathy: A hyperglycaemia- and insulin-resistance-induced heart disease. Diabetologia.

[39] Bugger H., Abel E. (2014). Molecular mechanisms of diabetic cardiomyopathy. Diabetologia.

[40] Nishida K., Otsu K. (2017). Inflammation and metabolic cardiomyopathy. Cardiovasc. Res..

[41] Sardu C., Pieretti G., D’Onofrio N., Ciccarelli F., Paolisso P., Passavanti M., Marfella R., Cioffi M., Mone P., Dalise A. (2018). Inflammatory Cytokines and SIRT1 Levels in Subcutaneous Abdominal Fat: Relationship with Cardiac Performance in Overweight Pre-diabetics Patients. Front. Physiol..

[42] D’Onofrio N., Pieretti G., Ciccarelli F., Gambardella A., Passariello N., Rizzo M., Barbieri M., Marfella R., Nicoletti G., Balestrieri M. (2019). Abdominal Fat SIRT6 Expression and Its Relationship with Inflammatory and Metabolic Pathways in Pre-Diabetic Overweight Patients. Int. J. Mol. Sci..

[43] Seferović P., Paulus W. (2015). Clinical diabetic cardiomyopathy: A two-faced disease with restrictive and dilated phenotypes. Eur. Heart J..

[44] Hölscher M., Bode C., Bugger H. (2016). Diabetic Cardiomyopathy: Does the Type of Diabetes Matter?. Int. J. Mol. Sci..

[45] Smiseth O. (2017). Evaluation of left ventricular diastolic function: State of the art after 35 years with Doppler assessment. J. Echocardiogr..

[46] Silbiger J. (2019). Pathophysiology and Echocardiographic Diagnosis of Left Ventricular Diastolic Dysfunction. J. Am. Soc. Echocardiogr..

[47] Mitter S., Shah S., Thomas J. (2017). A Test in Context. J. Am. Coll Cardiol.

[48] Lang M.R., Badano L.P., Mor-Avi V., Afilalo J., Armstrong A., Ernande L., Flachskampf F.A., Foster E., Goldstein S.A., Kuznetsova T. (2015). Recommendations for Cardiac Chamber Quantification by Echocardiography in Adults: An Update from the American Society of Echocardiography and the European Association of Cardiovascular Imaging. J. Am. Soc Echocardiogr.

[49] Kossaify A., Nasr M. (2019). Diastolic Dysfunction and the New Recommendations for Echocardiographic Assessment of Left Ventricular Diastolic Function: Summary of Guidelines and Novelties in Diagnosis and Grading. JDMS.

[50] Sanchis L., Andrea R., Falces C., Poyatos S., Vidal B., Sitges M. (2018). Differential Clinical Implications of Current Recommendations for the Evaluation of Left Ventricular Diastolic Function by Echocardiography. J. Am. Soc. Echocardiogr..

[51] Gottbrecht M., Salerno M., Aurigemma G. (2017). Evolution of diastolic function algorithms: Implications for clinical practice. Echocardiography.

[52] Fabiani I., Pugliese N., La Carrubba S., Conte L., Colonna P., Caso P., Benedetto F., Antonini-Canterin F., Citro R., Dini F. (2019). Interactive role of diastolic dysfunction and ventricular remodeling in asymptomatic subjects at increased risk of heart failure. Int. J. Cardiovasc. Imaging.

[53] Alonso-Gómez A., Tojal Sierra L., Fortuny Frau E., Goicolea Güemez L., Aboitiz Uribarri A., Portillo M., Toledo E., Schröder H., Salas-Salvadó J., Arós Borau F. (2019). Diastolic dysfunction and exercise capacity in patients with metabolic syndrome and overweight/obesity. Int J. Cardiol Heart Vasc.

[54] Shimron M., Williams L., Hazanov Y., Ghanim D., Kinany W., Amir O., Carasso S. (2018). Clinical and echocardiographic characteristics of patients in sinus rhythm, normal left ventricular function, and indeterminate diastolic function. Echocardiography.

[55] Fraser A. (2017). A manifesto for cardiovascular imaging: Addressing the human factor. Eur. Heart J. Cardiovasc. Imaging.

[56] Tamás É., Nylander E. (2018). Decision support for assessment of left ventricular diastolic function. Physiol. Rep..

[57] Bamaiyi A., Woodiwiss A., Peterson V., Gomes M., Libhaber C., Sareli P., Norton G. (2019). Insulin resistance influences the impact of hypertension on left ventricular diastolic dysfunction in a community sample. Clin Cardiol.

[58] Nagueh S. (2018). Classification of Left Ventricular Diastolic Dysfunction and Heart Failure Diagnosis and Prognosis. J. Am. Soc. Echocardiogr..

[59] Nagueh S., Abraham T., Aurigemma G., Bax J., Beladan C., Browning A., Chamsi-Pasha M., Delgado V., Derumeaux G., Dolci G. (2019). Interobserver Variability in Applying American Society of Echocardiography/European Association of Cardiovascular Imaging 2016 Guidelines for Estimation of Left Ventricular Filling Pressure. Circ. Cardiovasc. Imaging.

[60] Steeds R., Garbi M., Cardim N., Kasprzak J., Sade E., Nihoyannopoulos P., Popescu B., Stefanidis A., Cosyns B., Monaghan M. (2017). EACVI appropriateness criteria for the use of transthoracic echocardiography in adults: A report of literature and current practice review. Eur. Heart J. Cardiovasc. Imaging.

[61] Gori M., Canova P., Calabrese A., Cioffi G., Trevisan R., De Maria R., Grosu A., Iacovoni A., Fontana A., Greene S.J. (2017). Strategy to identify subjects with diabetes mellitus more suitable for selective echocardiographic screening: The DAVID-Berg study. Int. J. Cardiol..

[62] Blomstrand P., Sjöblom P., Nilsson M., Wijkman M., Engvall M., Länne T., Nyström F., Östgren C., Engvall J. (2018). Overweight and obesity impair left ventricular systolic function as measured by left ventricular ejection fraction and global longitudinal strain. Cardiovasc. Diabetol..

[63] Ng A., Prevedello F., Dolci G., Roos C., Djaberi R., Bertini M., Ewe S., Allman C., Leung D., Marsan N. (2018). Impact of Diabetes and Increasing Body Mass Index Category on Left Ventricular Systolic and Diastolic Function. J. Am. Soc. Echocardiogr..

[64] Loncarevic B., Trifunovic D., Soldatovic I., Vujisic-Tesic B. (2016). Silent diabetic cardiomyopathy in everyday practice: A clinical and echocardiographic study. BMC Cardiovasc. Disord..

[65] Ernande L., Bergerot C., Rietzschel E., De Buyzere M., Thibault H., PignonBlanc P., Croisille P., Ovize M., Groisne L., Moulin P. (2011). Diastolic Dysfunction in Patients with Type 2 Diabetes Mellitus: Is It Really the First Marker of Diabetic Cardiomyopathy?. J. Am. Soc. Echocardiogr..

[66] Cameli M., Mandoli G., Loiacono F., Dini F., Henein M., Mondillo S. (2015). Left atrial strain: A new parameter for assessment of left ventricular filling pressure. Heart Fail. Rev..

[67] Henein M.Y., Lindqvist P. (2015). Assessment of Left Ventricular Diastolic Function by Doppler Echocardiography. Card. Fail. Rev..

[68] D’Andrea A., Vriz O., Ferrara F., Cocchia R., Conte M., Di Maio M., Driussi C., Scarafile R., Martone F., Sperlongano S. (2018). Reference ranges and physiologic variations of left E/e’ ratio in healthy adults: Clinical and echocardiographic correlates. J. Cardiovasc. Echogr..

[69] van Grootel R., Menting M., McGhie J., Roos-Hesselink J., van den Bosch A. (2017). Echocardiographic chamber quantification in a healthy Dutch population. Neth. Heart J..

[70] Omar A., Narula S., Abdel Rahman M., Pedrizzetti G., Raslan H., Rifaie O., Narula J., Sengupta P. (2017). Precision Phenotyping in Heart Failure and Pattern Clustering of Ultrasound Data for the Assessment of Diastolic Dysfunction. JACC Cardiovasc. Imaging.

[71] Gong F., Campbell D., Prior D. (2017). Noninvasive Cardiac Imaging and the Prediction of Heart Failure Progression in Preclinical Stage A/B Subjects. JACC Cardiovasc. Imaging.

[72] Flachskampf F., Biering-Sørensen T., Solomon S., Duvernoy O., Bjerner T., Smiseth O. (2015). Cardiac Imaging to Evaluate Left Ventricular Diastolic Function. JACC Cardiovasc. Imaging.

[73] Plitt G., Spring J., Moulton M., Agrawal D. (2018). Mechanisms, diagnosis, and treatment of heart failure with preserved ejection fraction and diastolic dysfunction. Expert Rev. Cardiovasc..

[74] Davies M., D’Alessio D., Fradkin J., Kernan W., Mathieu C., Mingrone G., Rossing P., Tsapas A., Wexler D., Buse J. (2018). Management of hyperglycaemia in type 2 diabetes, 2018. A consensus report by the American Diabetes Association (ADA) and the European Association for the Study of Diabetes (EASD). Diabetologia.

[75] Sakamoto M., Matsutani D., Kayama Y. (2018). Possibility of a New Therapeutic Strategy for Left Ventricular Dysfunction in Type 2 Diabetes. J. Clin. Med. Res..

[76] McHugh K., DeVore A., Wu J., Matsouaka R., Fonarow G., Heidenreich P., Yancy C., Green J., Altman N., Hernandez A. (2019). Heart Failure with Preserved Ejection Fraction and Diabetes. JACC.

[77] de Lucia C., Sardu C., Metzinger L., Zuurbier C. (2019). Editorial: Diabetes and Heart Failure: Pathogenesis and Novel Therapeutic Approaches. Front. Physiol..

[78] Dunlay S., Givertz M., Aguilar D., Allen L., Chan M., Desai A., Deswal A., Dickson V., Kosiborod M., Lekavich C. (2019). Type 2 Diabetes Mellitus and Heart Failure: A Scientific Statement From the American Heart Association and the Heart Failure Society of America: This statement does not represent an update of the 2017 ACC/AHA/HFSA heart failure guideline update. Circulation.

[79] Arnett D., Blumenthal R., Albert M., Buroker A., Goldberger Z., Hahn E., Himmelfarb C., Khera A., Lloyd-Jones D., McEvoy J. (2019). 2019 ACC/AHA Guideline on the Primary Prevention of Cardiovascular Disease. Circulation.

[80] Rossignol P., Hernandez A., Solomon S., Zannad F. (2019). Heart failure drug treatment. Lancet.

[81] Selmeryd J., Henriksen E., Leppert J., Hedberg P. (2016). Interstudy heterogeneity of definitions of diastolic dysfunction severely affects reported prevalence. Eur. Heart J. Cardiovasc. Imaging.

[82] Bouthoorn S., Valstar G., Gohar A., den Ruijter H., Reitsma H., Hoes A., Rutten F. (2018). The prevalence of left ventricular diastolic dysfunction and heart failure with preserved ejection fraction in men and women with type 2 diabetes: A systematic review and meta-analysis. Diab. Vasc. Dis. Res..

[83] Reddy Y., Carter R., Obokata M., Redfield M., Borlaug B. (2018). A Simple, Evidence-Based Approach to Help Guide Diagnosis of Heart Failure with Preserved Ejection Fraction. Circulation.

[84] Sardu C., Marfella R., Santamaria M., Papini S., Parisi Q., Sacra C., Colaprete D., Paolisso G., Rizzo M., Barbieri M. (2018). Stretch. Injury and Inflammation Markers Evaluation to Predict Clinical Outcomes After Implantable Cardioverter Defibrillator Therapy in Heart Failure Patients with Metabolic Syndrome. Front. Physiol..

[85] Meagher P., Adam M., Civitarese R., Bugyei-Twum A., Connelly K. (2018). Heart Failure with Preserved Ejection Fraction in Diabetes: Mechanisms and Management. Can. J. Cardiol..

